# The gut microbiome: a vital link to hyperuricemia, gout and acute flares?

**DOI:** 10.3389/fendo.2025.1643566

**Published:** 2025-08-08

**Authors:** Wei Wang, Liping Wang, Jing Chen, Xinyi Yang, Qingyu Guo, Zhen Zhang, Jingjing Liang, Ping Gu, Jiaqing Shao

**Affiliations:** ^1^ Department of Endocrinology, Jinling Hospital, Affiliated Hospital of Medical School, Nanjing University, Nanjing, Jiangsu, China; ^2^ Department of Endocrinology, Zibo Central Hospital, Zibo, Shandong, China; ^3^ Department of Endocrinology, Jinhu County People’s Hospital, Jiangsu, China; ^4^ Department of Endocrinology, Jinling Hospital, the First School of Clinical Medicine, Southern Medical University, Shenzhen, Guangdong, China

**Keywords:** gout, hyperuricemia, gut microbiota, acute gout, asymptomatic hyperuricemia

## Abstract

**Objectives:**

To explore the associations between the gut microbiome and asymptomatic hyperuricemia, as well as acute gout flares.

**Methods:**

Forty-three Chinese participants were divided into healthy and hyperuricemic groups according to serum uric acid (SUA) levels. The hyperuricemia group were further separated into asymptomatic hyperuricemia (HUA) and gout patients on the basis of their clinical symptoms. In addition, the gout group was further divided into intercritical gout and acute gout groups on the basis of the claim of joint pain and relevant clinical parameters. 16S rRNA sequencing was used to evaluate the microbiome composition of all the groups.

**Results:**

A dramatic decreasing trend in microbial richness and diversity was observed in hyperuricemic patients compared with healthy controls. The same decreasing trend in microbial relative abundance was also observed. The butyrate-producing genera Faecalibacterium, Coprococcus and Enterococcus were markedly decreased in hyperuricemic patients. Moreover, opportunistic pathogens, such as the phylum Proteobacteria and genus Fusobacterium, were enriched in the hyperuricemia group. Furthermore, the gut microbiota of gout patients also exhibited significantly reduced microbial diversity compared with asymptomatic hyperuricemic patients, characterized by decreased richness of the genera Dialister, Ruminococcus, and Faecalibacterium. Greater differences in microbial richness and diversity can still be observed when gout flares occur. The abundances of Bacteroides and Lachnospira genera decreased in the acute gout stage.

**Conclusion:**

Our study revealed that community richness and diversity change during the process of gout or HUA, especially during acute gout flares. Metagenomic species were significantly altered during different stages of hyperuricemia.

## Highlights

- Microbial richness and diversity were significantly lower in hyperuricemic patients than in healthy controls.
- Compared with the asymptomatic hyperuricemic group, the gout group was positively associated with alterations in the gut microbiota at both the phylum and genus levels.
- The abundance of some metagenomic species that produce short-chain fatty acids (SCFAs) increases significantly during acute gout attack.

## Introduction

Gout, which is a complex disorder of purine metabolism, has become a major public health issue worldwide, especially in Oceanian countries. Hyperuricemia (HUA) is the most important risk factor for the development of gout as the deposition of monosodium urate (MSU) crystals in the joints results in an acute inflammatory response. Rapid economic development has led to significant changes in global lifestyle and dietary patterns, including increased consumption of purine-rich foods and alcohol (1). The prevalence of gout and hyperuricemia increases with increasing age, and the incidence of these diseases is increasing (2). As the world’s largest developing country, China has experienced a significant shift toward Western-style dietary patterns. Epidemiological studies indicate this transition has contributed to rising prevalence rates of hyperuricemia and gout. A systematic meta-analysis revealed that the prevalence of gout in mainland China was 0.9% from 2000–2005, while this number increased to 1.1% from 2006–2010 and 1.4% from 2011–2014 (3, 4). According to two meta-analyses in mainland China, the estimated prevalence of hyperuricemia was 17.4% in 2020, which was higher than the prevalence in 2014 (13.3%) (5). An alarming increase in the overall prevalence of hyperuricemia in the Chinese adult population was observed, ranging from 11.1% to 14.0% over three years (from 2015–16 to 2018-19) (6). Several analyses have revealed causal associations between hyperuricemia and type 2 diabetes, hypertension, stroke, cardiovascular events or heart failure (7). Furthermore, large prospective studies have shown that gout is associated with an increased risk of death and causally contributes to worse outcomes (8). Camilla et al. highlighted that WHO projections suggest that gout mortality may increase by 55% by 2060 (9). Therefore, effective measures should be adopted to prevent the spread of these diseases.

Gout is defined by the presence of MSU crystals in the joints, which results from chronic elevation of serum uric acid (SUA) levels above the saturation point. A sustained high level of SUA known as hyperuricemia is an essential step in the development of gout. Prolonged hyperuricemia can lead to the formation of MSU crystals that accumulate in joints and other tissues. Serum urate levels exhibit a concentration-dependent association with the incidence of gout (10). The dominant cause of hyperuricemia is an imbalance between uric acid production and excretion, which means that decreased excretion, increased synthesis or both lead to elevated serum uric acid levels (11). Currently, the most commonly used urate-lowering therapy (ULT) is focused on inhibiting the production of uric acid (12). In addition to renal excretion, urate is also excreted via the gut and is further metabolized by resident bacteria (13). Elevated serum uric acid (SUA) levels are increasingly implicated in the development of chronic kidney disease (CKD) and the progression of established renal dysfunction (13). Therefore, intestinal elimination of uric acid excretion is an important alternative pathway during renal insufficiency. Nevertheless, there have been few mechanistic studies on the extrarenal excretion pathway of uric acid.

Hyperuricemia represents the primary risk factor for gout. However, epidemiological studies indicate that the majority of individuals with hyperuricemia remain asymptomatic throughout their lifetime; only approximately 10% progress to clinically evident gout (14). One-third of patients have normal SUA levels during acute flares of gouty arthritis. Interestingly, the proportion of MSU deposits in patients with early clinical gout (one or two joint flares) seems similar to that in asymptomatic hyperuricemic patients according to ultrasound scans (15). Thus, it is difficult to predict gout attack by monitoring the uric acid level or deposits of MSU crystals, and more factors that have not yet been studied should be considered.

Previous studies have shown that gastrointestinal dysbiosis plays a significant role in the development of metabolic diseases, such as type 2 diabetes (T2DM), hypertension, hyperuricemia and gout (16, 17). In recent years, some progress has been achieved in research investigating the relationship between gut microbes and HUA and gout. The intestinal microbiota of individuals with gout exhibits significant compositional and functional differences compared to that of healthy controls (18). The Microbial Index of Gout was proposed as a new monitoring tool for the diagnosis of gout and achieved a higher accuracy of 88.9% than conventional blood uric acid tests (19). An increasing number of studies have shown that the gut microbiota may modulate local immune responses in mice and that the human gut microbiota is linked to inflammatory cytokine production (20, 21). However, few studies have examined the association between the gut microbiota and hyperuricemia in humans.

Therefore, we investigated whether the influence of the gut microbiota extends beyond the confines of the gut and whether the gut microbiota plays an essential role in the development of hyperuricemia and gout, especially in the process of acute gout. To achieve these initial goals, we devised a cross-sectional study based on Illumina MiSeq sequencing of the 16S rRNA gene extracted from stool samples from a 43-member Chinese cohort.

## Methods

### Study participants

The clinical trial included 43 participants receiving inpatient or outpatient therapies from the Department of Endocrinology at Jingling Hospital. All participants were non-smoking Chinese adult male volunteers aged 20–65 years. Subjects were excluded if they had suffered from the following disorders in the past: hypertension, lipid metabolism disorders, diabetes, carbohydrate metabolism disorders, and gastrointestinal diseases such as diarrhea, dysentery, constipation, and other conditions needing medication. All participants were required to have no documented history of hazardous drinking or smoking at baseline. Additionally, they were required to abstain from consuming antibiotics, probiotics/prebiotics, alcohol, and purine-rich foods (e.g., seafood, organ meats, red meat, yeast extracts) for 1 month before sample collection. These patients also had no family genetic disorders. This study was approved by the Ethics Committee of Nanjing Jinling Hospital, and all participants provided written informed consent.

The participants were divided into healthy and hyperuricemic groups (the SUA level gradually increased to more than 420 μmol/L). On the basis of the European League Against Rheumatism (EULAR) guidelines updated in 2018, the hyperuricemia group, which consists of 34 adult patients, was further divided into asymptomatic HUA patients (people have hyperuricemia without MSU crystals and symptoms of gout) and gout patients (people have MSU crystal deposition and clinical disease elements such as gout flare, chronic gouty arthritis and tophi) (22). In addition, the gout group was further divided into intercritical gout and acute gout groups on the basis of the claim of joint pain and relevant clinical parameters. All the subjects were diagnosed by the treating endocrinologists from the subject’s history and available laboratory data and provided informed consent to participate in the study.

### Sample collection and storage

Blood and stool samples were collected in the morning within 2 h. These blood samples were isolated and stored at −80°C for detection of SUA, creatinine (Cr), fast plasma glucose (FPG), glutamic oxaloacetic transaminase (AST), alanine aminotransferase (ALT), total triglyceride (TG), cholesterol (CHOL), high-density lipoprotein cholesterol (HDLC) and low-density lipoprotein cholesterol (LDLC) levels. The above serum levels were measured via a Siemens-ADVIA2400 automatic biochemistry analyzer. Bacterial genomic DNA was extracted from the stool samples according to the instructions of the Magen Hipure Soil DNA Kit and the Qubit dsDNA HS Assay Kit. Total DNA was also isolated and stored at −80°C for Illumina MiSeq sequencing analysis (23).

### 16S rRNA gene amplification and sequencing

A fusion primer set that incorporates adapter sequences, indexing barcodes and a PCR primer (515F: GTGCCAGCMGCCGCGGTAA, 806R: GGACTACHVGGGTWTCTAAT) (Caporaso JG, 2010PNAS) was used to amplify the V4 region of the 16S rRNA gene. The PCR products were subsequently checked for size and specificity via agarose gel electrophoresis and subsequently purified. Finally, the amplicons were pooled at equimolar ratios and sequenced via the Ion PGM Hi-Q 400 Kit at the core facility of the Suzhou Institute of Systems Medicine (ISM). After sequencing, the reads were filtered to remove low-quality and polyclonal sequences, and adaptor reads were trimmed. The filtered data were further compared with the Gold database, and the chimera reads were detected via Usearch (Version 8.1.1861). The resulting reads for each sample were clustered into operational taxonomic units (OTUs) at the level of 97% similarity via QIIME (version 1.9.1). A representative sequence for each OTU was selected, and the taxonomic information was annotated via QIIME (version 1.9.1) and the Silva database (release 119). The sequencing reaction was conducted by Suzhou Geneworks Technology Co., Ltd., Jiangsu, China (24).

### Calculations and statistical analysis

Normally distributed variables were performed using unpaired t tests (25). Abnormally distributed variables were analyzed via nonparametric (Kruskal-Wallis) tests, with significance set at p< 0.05 unless otherwise specified (26). To compare participant characteristics(age, BMI, blood pressure, ALT, AST, FDG and blood lipids) of two groups, the unpaired t tests was used. Differences in alpha indices and the composition structure of gut microbiota were evaluated with Kruskall-Wallis tests. In the figures and tables, the data are presented as the means ± SEMs, and p<0.05 was considered significant. GraphPad Prism software versions 5 and R (version 4.2.3) were used for all the statistical analyses.

## Results

### Alterations in the gut microbiota between hyperuricemic patients and healthy controls

We classified 43 volunteers into subgroups by SUA level. The healthy group (SUA level less than 420 µmol/L), which consisted of 9 healthy male adults, served as controls. The hyperuricemia group consisted of 34 adult patients (with SUA levels greater than 420 µmol/L), including asymptomatic hyperuricemia and gout patients. The clinical characteristics of these volunteers are presented in **Table 1**. There were no statistically significant differences in age, BMI, blood pressure, ALT, AST, FDG or blood lipid metabolic levels between the two groups (p>0.05, unpaired t test, **Table 1**), whereas significant differences were detected in the serum uric acid and creatinine levels (p=0.000 and p=0.019, respectively; unpaired t test, **Table 1**).

**Table 1:** Differences in the baseline characteristics between healthy people and hyperuricemic patients.

Characteristics	Control (n=9)	HU (n=34)	p Values
Age (years)	40.20 ± 15.29	41.65 ± 12.86	0.610
SBP (mmHg)	125.10 ± 3.72	124.38 ± 5.28	0.815
DBP (mmHg)	74.40 ± 6.39	76.94 ± 5.82	0.249
BMI (kg/m²)	23.81 ± 1.94	25.25 ± 2.27	0.062
UA (μmol/L)	335.50 ± 48.79	568.76 ± 85.67	0.000*
Cr (μmol/L)	65.40 ± 14.97	75.91 ± 11.20	0.019*
ALT (U/L)	5.88 ± 3.06	14.08 ± 11.19	0.151
AST (U/L)	9.14 ± 2.11	15.53 ± 8.24	0.066
FPG (mmol/L)	4.90 ± 0.60	4.92 ± 0.72	1.000
CHOL (mmol/L)	4.62 ± 0.59	4.57 ± 0.65	0.710
TG (mmol/L)	1.55 ± 0.65	1.77 ± 0.66	0.273
HDLC (mmol/L)	1.08 ± 0.32	0.98 ± 0.17	0.312
LDLC (mmol/L)	2.89 ± 0.77	2.80 ± 0.63	0.629

HU: All the patients included asymptomatic HUA and gout patients; SBP represents systolic blood pressure, and DBP represents diastolic blood pressure. The symbol (*) denotes statistical significance where p-values are less than 0.05.

**Figure 1:**
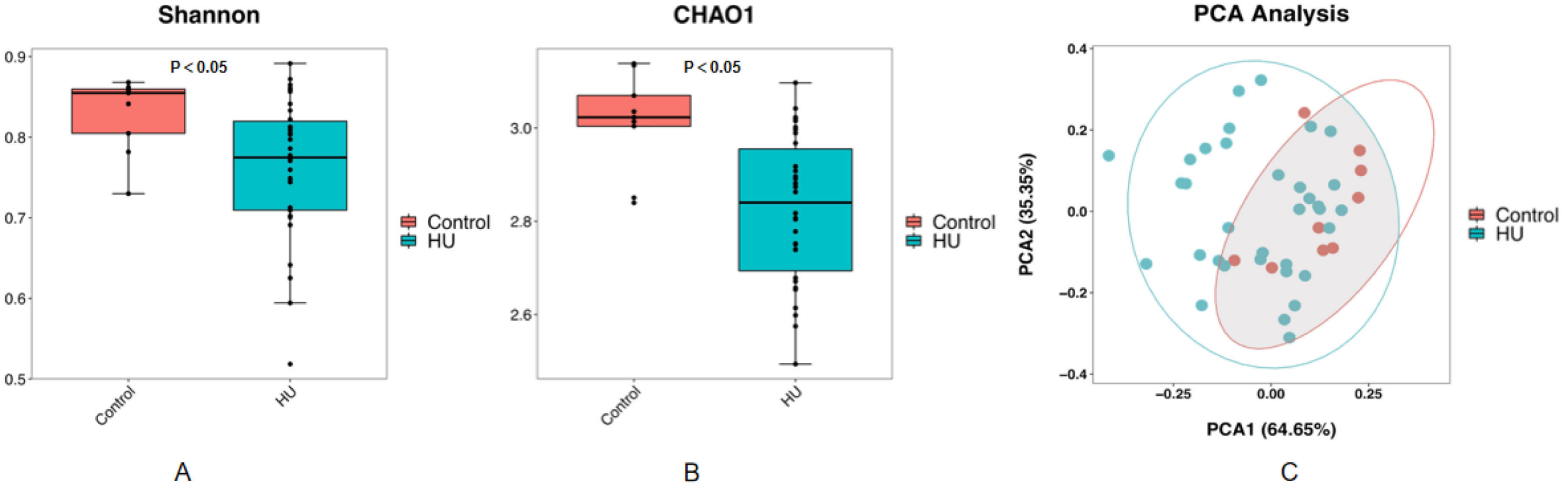
The community richness and diversity of the gut microbiota decreased profoundly in hyperuricemic patients compared with healthy controls. Chao1 **(A)** and Shannon **(B)** indices are both indices of alpha diversity, and PCoA score plots **(C)** based on the unweighted UniFrac distance of all participants showed the gut microbiota composition was different between the hyperuricemic group and the control group.

To evaluate whether any difference in community richness or diversity of the gut microbiota was detected, we used the Chao1(community richness) (27) and Shannon(community diversity) (28) indices to analyze the alpha diversity of all the participants The gut microbiota of the hyperuricemic group was significantly less rich and diverse (p < 0.05, Kruskal–Wallis tests, **Figures 1A, B**) than that of the control group. PCA is an index of beta diversity that uses the unweighted UniFrac distances of 16S rRNA sequence profiles. The points that represented the composition of the intestinal microbiota of all participants formed two clusters (**Figure 1C**). PC1 and PC2 explained 64.65% and 35.35% of the total variance, respectively. A significant separation (P < 0.001, Kruskal–Wallis tests, **Figure 1C**) was observed for PC 1.

For further analysis, the composition and structure of the intestinal flora were profoundly altered at the phylum and genus levels, as shown in **Supplementary Figure S1**. At the phylum level, the histogram (**Supplementary Figure S1**) revealed that the dominant bacteria of all groups were Firmicutes, Bacteroidia, Proteobacteria, Actinobacteria and Fusobacteria, but the percentage of the gut microbiome changed. Compared with those in the control group, Proteobacteria were significantly greater in hyperuricemic patients (p=0.042, Kruskal–Wallis test, **Supplementary Figure S1**) at the phylum level. Furthermore, we also analyzed the overall composition of the microbiota and average relative abundance at the genus level. Among the hyperuricemic subjects, the abundances of Faecalibacterium, Coprococcus, Enterococcus and Prevotella were significantly lower (p=0.034, p=0.001, p=0.017, p=0.001, respectively, Kruskal–Wallis test, **Supplementary Figure S2**) than those in healthy controls, and these bacteria are known to be vital for healthy intestinal microbial homeostasis. Moreover, the significant increase in Fusobacterium in the hyperuricemia group also needs our attention (p =0.031, Kruskal–Wallis test, **Supplementary Figure S2**). These transitions of the gut microbiome might play a potential role in the pathogenesis of hyperuricemia.

### Changes in microbial richness, diversity and composition in the gout group compared with the asymptomatic hyperuricemia group

According to the EULAR guidelines updated in 2016, asymptomatic hyperuricemia was regarded as the initial state preceding acute gout flares. However, epidemiologic studies have demonstrated that the chance of developing gout in people with hyperuricemia is fairly low. Therefore, we divided the 34 hyperuricemic patients into two groups. We chose 10 patients whose SUA levels were more than 420 µmol/L but never had symptoms or signs of urate crystal deposition as the asymptomatic hyperuricemia group, while the other patients composed the gout group.

**Figure 2:**
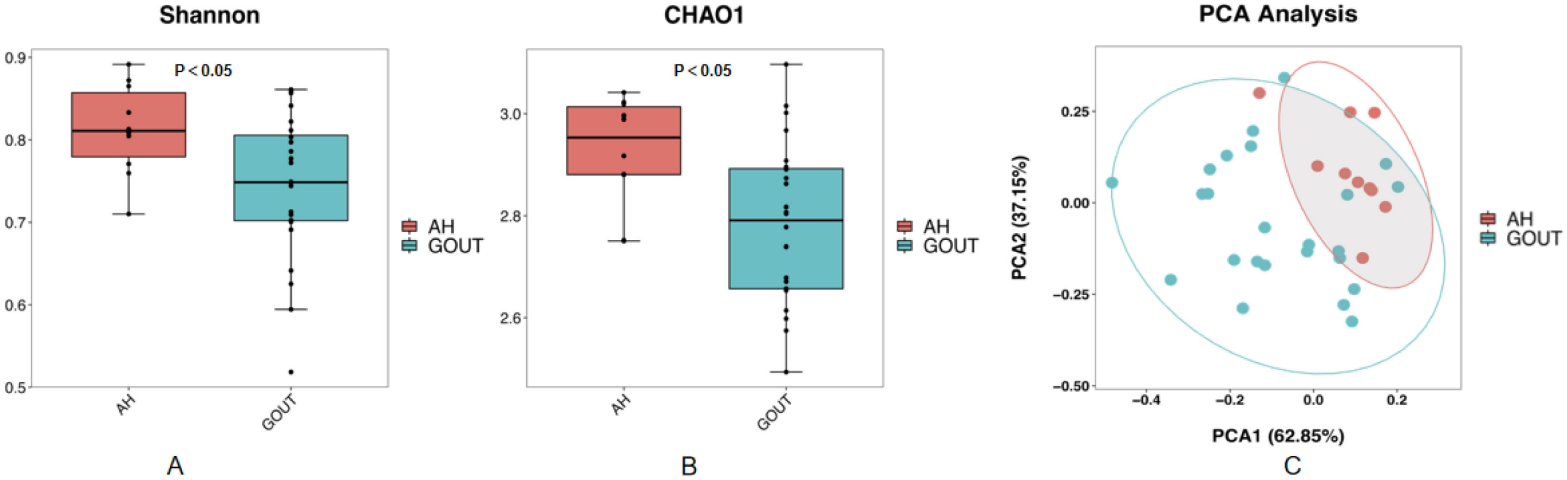
Compared with those in asymptomatic HUA patients, the community richness and diversity of the gut microbiota in gout patients are profoundly decreased. Chao1 **(A)** and Shannon **(B)** indices both decreased in gout patients compared with asymptomatic hyperuricemic patients. PCoA score plots **(C)** based on the unweighted UniFrac distance showed that the gut microbiota composition was different between asymptomatic hyperuricemic patients and gout patients.

We also analyzed the basic characteristics of the 34 patients included in the study, and the results are provided in **Table 2**. Similarly, no significant differences were detected in the measures of age, BMI, blood pressure, ALT, AST, FDG, blood lipids or creatinine (p>0.05, unpaired t test, **Table 2**), except for the serum uric acid level (P=0.00, unpaired t test, **Table 2**). The alpha diversity of the intestinal microbiota was still significantly different between asymptomatic hyperuricemic patients and gout patients. The microbial richness and diversity of the gout group were both significantly lower (p < 0.05, Kruskal–Wallis tests, **Figures 2A, B**) than those of the asymptomatic hyperuricemia group. Moreover, PC1 and PC2 of the beta diversity explained 62.85% and 37.15% of the total variance, respectively (**Figure 2C**).

**Table 2:** Differences in baseline characteristics between asymptomatic HUA patients and gout patients.

Characteristics	AH (n=10)	Gout (n=24)	p Values
Age (years)	38.80 ± 16.31	42.83 ± 11.32	0.564
SBP (mmHg)	124.30 ± 6.99	124.42 ± 4.56	0.912
DBP (mmHg)	73.20 ± 6.23	78.50 ± 4.97	0.068
BMI (kg/m²)	24.42 ± 2.61	25.59 ± 2.07	0.060
UA (μmol/L)	534.60 ± 82.19	583.00 ± 84.66	0.000*
Cr (μmol/L)	77.30 ± 8.35	75.33 ± 12.31	0.057
ALT (U/L)	18.34 ± 14.77	12.31 ± 9.13	0.301
AST (U/L)	16.49 ± 10.18	15.13 ± 7.51	0.185
FPG (mmol/L)	5.31 ± 0.56	4.76 ± 0.72	0.117
CHOL (mmol/L)	4.15 ± 0.82	4.74 ± 0.49	0.148
TG (mmol/L)	1.41 ± 0.59	1.92 ± 0.64	0.057
HDLC (mmol/L)	0.94 ± 0.16	1.01 ± 0.18	0.487
LDLC (mmol/L)	2.64 ± 0.58	2.87 ± 0.65	0.451

AH: asymptomatic HUA patients; Gout: all gout patients, including those in the acute and resolution stages; SBP represents systolic pressure, and DBP represents diastolic pressure. The symbol (*) denotes statistical significance where p-values are less than 0.05.

Differences in the main composition of the gut microbiome between the two groups are presented in **Supplementary Figure S1**. The gout group also presented a significantly greater relative abundance of Proteobacteria (p =0.016, Kruskal–Wallis test, **Supplementary Figure S1**) at the phylum level than did the asymptomatic hyperuricemic group according to the histogram of the intestinal flora. In addition, LEfSe analysis revealed that the abundances of Faecalibacterium, Dialister and Ruminococcus in the gout group were significantly lower (p=0.034, p=0.018, and p=0.006, respectively, Kruskal–Wallis test, **Supplementary Figure S3**) than those in the asymptomatic hyperuricemic group at the genus level. However, the relative abundance of Dorea significantly increased (p =0.005, Kruskal–Wallis test, **Supplementary Figure S3**) in gout patients.

### Differences in the gout microbiome in different phases of gout

The progression of gout can be defined by different pathophysiological stages. Patients with MSU deposition develop an acute inflammatory response in joints, such as swelling, heat, redness, and difficulty moving the affected joint, manifesting as acute gout flares. After initial presentation with an acute flare, the symptoms resolve entirely, which is called the resolution stage and is also known as intercritical gout. We further divided the gout group, which consisted of 24 adult patients, into intercritical gout and acute gout groups on the basis of the claim of joint pain and relevant clinical parameters.

**Figure 3:**
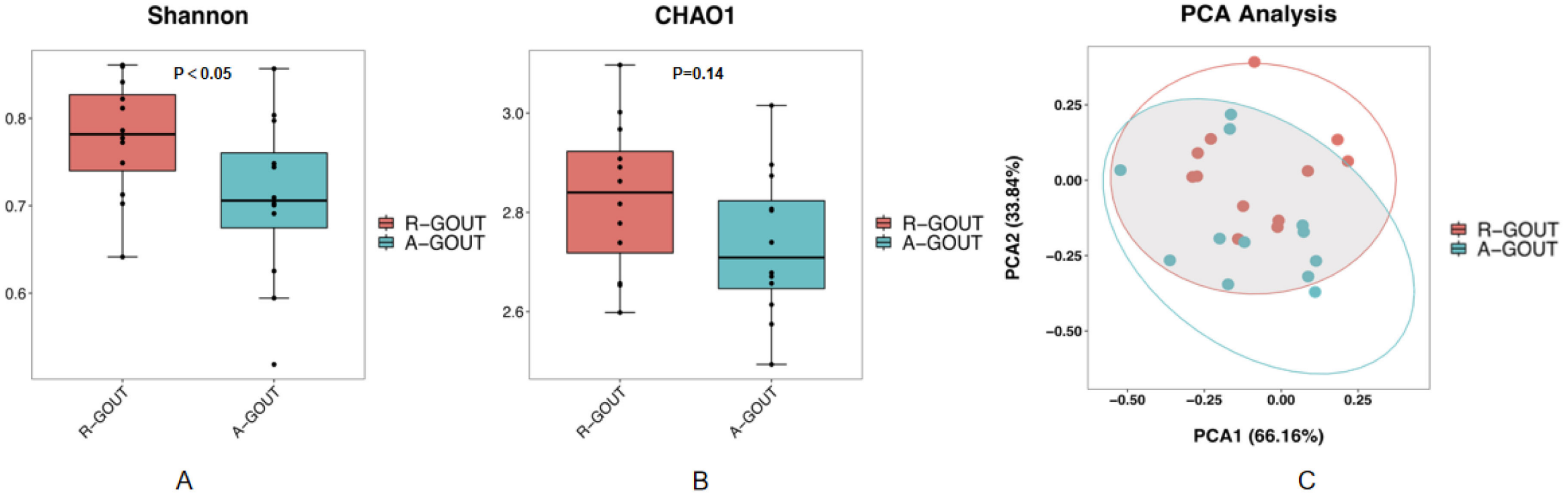
The community richness and diversity of the gut microbiota decreased profoundly in acute gout patients. The values of the Chao1 index **(A)** and Shannon index **(B)** were significantly lower in patients with acute gout patients than in intermediate gout group. These findings suggest that acute gout is associated with decreased α-diversity. PCoA score plots **(C)** based on the unweighted UniFrac distance showed that the gut microbiota composition was different between the intermediate gout group and the acute gout group.

There was no evidence of significant differences in all the basic characteristics, including serum uric acid and creatinine, between the intermediate gout group and the acute gout group (p>0.05, unpaired t test, **Table 3**). Both the Chao1 index and the Shannon index were lower (p < 0.05, Kruskal–Wallis tests, **Figures 3A, B**) in the acute gout group than in the gout group in the resolution stage. PCA was also performed, and significant alterations were still observed, with 66.16% PC1 and 33.84% PC2 of the total variance (**Figure 3C**).

**Table 3:** The baseline characteristics of gout patients in the resolution and acute stages.

Characteristics	R-Gout (n=12)	A-Gout (n=12)	p Values
Age (years)	43.75 ± 11.55	41.92 ± 11.52	0.843
SBP (mmHg)	125.25 ± 4.35	123.58 ± 4.81	0.378
DBP (mmHg)	78.83 ± 5.44	78.17 ± 4.68	0.799
BMI (kg/m²)	25.17 ± 2.15	26.02 ± 1.98	0.514
UA (μmol/L)	558.42 ± 89.01	607.58 ± 75.79	0.060
Cr (μmol/L)	73.58 ± 10.17	77.08 ± 14.37	0.551
ALT (U/L)	13.34 ± 10.10	11.29 ± 8.36	0.671
AST (U/L)	15.06 ± 8.16	15.20 ± 7.16	0.932
FPG (mmol/L)	4.79 ± 0.46	4.74 ± 0.94	0.713
CHOL (mmol/L)	4.67 ± 0.49	4.82 ± 0.50	0.378
TG (mmol/L)	1.91 ± 0.68	1.94 ± 0.63	1.000
HDLC (mmol/L)	0.98 ± 0.20	1.03 ± 0.15	0.410
LDLC (mmol/L)	2.83 ± 0.74	2.91 ± 0.58	0.843

R-GOUT: Gout patients in the resolution stage; A-GOUT: Patients in acute gout; SBP represents systolic pressure, and DBP represents diastolic pressure.

As shown in **Supplementary Figure S1**, we detected few differences in the gut bacterial populations between the intermediate gout stage and acute arthritis stage at the phylum level. The acute gout group presented an increased abundance of the phylum Actinobacteria (p =0.048, Kruskal–Wallis test, **Supplementary Figure S1**) and a decreased abundance of Bacteroides (p =0.089, Kruskal–Wallis test, **Supplementary Figure S1**), although these differences did not reach significance. At the genus level, the acute flare group presented a lower proportion of Bacteroides and Lachnospira (p=0.033 and p=0.039, respectively; Kruskal–Wallis test; **Supplementary Figure S4**).

### Potential correlation between the gut microbiota and UA level

Spearman’s correlation coefficient analysis was used to explore the potential correlation between different bacterial abundances and clinical characteristics. Unexpectedly, there was no correlation between serum uric acid or creatinine levels and the relative abundance of the gut microbiome at the phylum level in any of the participants, as shown in **Supplementary Figure S5**. At the genus level, a heatmap revealed that uric acid was negatively correlated with the relative abundance of Coprococcus (**Supplementary Figure S6**).

In addition, BMI and TG were negatively correlated with the relative abundances of Faecalibacterium and Ruminococcus (**Supplementary Figure S6**) and positively correlated with the relative abundance of Proteobacteria (**Supplementary Figure S5**).

## Discussion

During the past two decades, observational findings have suggested that the gut microbiota is closely linked to multiple systemic diseases, such as cardiovascular diseases and metabolic syndrome. Recent evidence suggests that the intestinal microbiota in gout patients is very distinct from that in healthy individuals. These findings demonstrated that the gut microbiota may be related to gout. Furthermore, studies suggest that there are reduced levels of short-chain fatty acids (SCFAs), reduced inflammasome assembly, and reduced inflammatory responses to MSU crystals in germ-free mice (29, 30). However, few studies have indicated a direct association between hyperuricemia and the gut microbiome in human disease, especially between advanced gout and acute gout.

Therefore, we sequenced the total bacterial DNA of stool samples from a cohort of 43 Chinese individuals. Gene amplification and sequencing revealed that the community richness or diversity of the gut microbiota profile was significantly altered in patients with HUA compared with healthy controls, which was in agreement with some previous findings. In addition, we observed that the composition of the gut flora in asymptomatic hyperuricemic patients was quite different from that in gout patients. In particular, community richness or diversity decreased more when acute gout flared. Decreased richness or diversity of the gut microbiome can cause a number of physiological disorders in the host, according to previous studies. Therefore, we believe that decreases in and changes in bacterial diversity, richness and composition may be closely related to the progression of gout.

At the phylum level, several phyla, such as Proteobacteria, were observed to increase in hyperuricemia patients. Recent studies have demonstrated that an increased prevalence of Proteobacteria can cause instability of the microbial community under abnormal metabolic conditions (31). An altered gut microbial community accompanying the relative abundance of Proteobacteria was observed not only in acute inflammation but also in chronic inflammation, as supported by studies of mice deficient in both the innate and adaptive immune systems (32). An uncontrolled increase in Proteobacteria can further facilitate inflammation or invasion by inducing proinflammatory interleukin-17 (IL-17) or transforming growth factor-β (TGF-β) production (33). Interestingly, our research revealed a significantly greater level of Proteobacteria in gout patients than in asymptomatic hyperuricemic patients. Although hyperuricemia has been identified as the key risk factor for gout and gouty arthritis, only 10% of these patients eventually develop gout. The most well-recognized mechanism of gout is that when the whole body is in an increased serum urate concentration state, MSU crystals activate the innate immune response by triggering inflammasome pathways, resulting in the release of proinflammatory cytokines. Therefore, we believe that the increase in Proteobacteria may be closely related to the progression of gout by triggering inflammatory responses.

At the genus level, we found that there was a lower relative abundance of Faecalibacterium, Coprococcus, and Enterococcus in hyperuricemic patients. In particular, the abundance of Faecalibacterium was lower in gout patients than in asymptomatic hyperuricemic patients. Faecalibacterium is a butyrate-producing bacterium with anti-inflammatory and protective effects against obesity, diabetes mellitus and hypertension (34, 35). Recent efforts imply that butyrate plays critical protective roles in maintaining human gut health. Butyrate is the major source of energy to the colonic mucosa and is an important regulator of gene expression, immunity, differentiation and apoptosis in host cells (36). Increasing evidence indicates that several genera, such as Faecalibacterium, are altered in hyperuricemia model rats and gout patients (37). A recent study reported that SCFA depletion in gout may result in increased local and systemic inflammation through the modification of their host receptors (38). In addition, Guo reported a remarkable reduction in Faecalibacterium abundance in gout and T2DM patients, which is in line with our findings. Taken together, these findings suggest that the abundance of Faecalibacterium may be a potential protective indicator of intestinal health.

Coprococcus is also a butyrate-producing bacterium. In our study, we showed that the abundance of the Coprococcus genus was greater in healthy controls than in hyperuricemic patients. Most importantly, Corprococus was closely linked to the SUA level according to the correlation heatmap. These results are similar to those of many previously reported studies. A limited number of studies have shown a significant reduction in Coprococcus in hypertensive patients and rats (39, 40). Moreover, recent research has shown that Coprococcus abundance is lower in the intestinal microbiota of gout patients, which is in line with our findings (41). In a large rural community-based cohort, hyperuricemia was related to a low relative abundance of the genus Coprococcus (42). Overall, these results suggest that decreased Coprococcus may contribute to dysfunction in uric acid degradation.

Previous studies revealed that butyrate-producing bacteria may provide ATP for intestinal epithelial cells to excrete UA through ATP-binding cassette superfamily G member 2 and solute carrier protein 2 family member 9 in male gout patients (43). Our observations are consistent with these reports, and together with findings from the present study, butyrate appears to play a role in uric acid regulation. Further studies are needed to assess whether butyrate supplementation has an important role in reducing the incidence of hyperuricemia and gout.

In addition, our study revealed that Fusobacteria was significantly elevated in hyperuricemic patients. Certain Fusobacteria species may be considered opportunistic pathogens in the human gut. Metagenomic studies have shown that many diseases, including T2DM, colon cancer and rheumatoid arthritis, are associated with intestinal dysbiosis characterized by the enrichment of Fusobacterium (44–46). It can induce the expression of proinflammatory cytokines, including IL-6, IL-8 and NF-κB. These findings suggest that enrichment of the Fusobacterium may contribute to gut barrier dysbiosis and hyperuricemia via regulation of the expression of inflammatory factors.

Compared with those associated with intercritical gout, we detected an increased relative abundance of Actinobacteria and a decreased abundance of Bacteroides in terms of phyla when gout flares occurred. The bacteria most represented in the human gut of Actinobacteria are Bifidobacteria, which produce high concentrations of acetate. Previous studies have shown that a high concentration of Actinobacteria is positively correlated with improved glucose homeostasis and reduced obesity and inflammation. The genus Bacteroides is considered to produce propionate. Many studies have shown that the abundance of Bacteroides is increased in many diseases, such as rheumatoid arthritis and systemic lupus erythematosus.

Gut microbiota as a potential therapeutic target in HUA and gout has become a new research hotspot.Recent studies have shown that probiotic treatment and fecal microbiota transplantation (FMT) experiments can play a role in the treatment of HUA and gout by altering the structure of the gut microbiota (47). Regarding probiotic treatment, a study found that Lactobacillus hammocks(LGG) could alleviate HUA by regulating nucleoside and proline metabolism in goose (48). Limos lactobacillus fermentum JL-3 can regulate the gut microbiota imbalance caused by HUA and restore some inflammatory markers and oxidative stress indicators associated with HUA (49). Evidence indicates that prebiotics such as anserine and chicory ameliorate hyperuricemia primarily through gut microbiota modulation, with anserine mediating protective effects against renal inflammation and chicory alleviating the LPS/TLR4 axis (50). Recent human studies link gut microbiome disruption to gout pathogenesis. A large retrospective cohort analysis associating prior clindamycin (vs.trimethoprim/sulfamethoxazole) exposure with a ~30% increased incident gout risk, and the controlled Food and Resulting Microbial Metabolites (FARMM) trial demonstrating that antibiotic-induced microbiota depletion rapidly elevates fecal urate by 40-50% and reduces key purine-degrading bacterial gene abundance, particularly under a synthetic diet (51). The above studies suggest that dietary modifications and probiotic supplementation may emerge as an effective UA-lowering strategy. Furthermore, FMT experiments have demonstrated that transplanting the gut microbiota can reduce the serum UA levels in gout patients, is associated with a reduction in the frequency and duration of acute gout flares (52). These results currently provides more treatment modes for patients with HUA and gout.

In addition, a mendelian radomization analysis showed that the genus Ruminococcus was linked to a lower risk of HUA, while the family Clostridiaceae was associated with a higher risk of HUA. Clinical validation showed that high Clostridiaceae and low Ruminococcus abundance were associated with a higher risk of HUA, and the predictive diagnostic efficacy of Clostridiaceae was better (53).These results collectively suggest that gut microbiota may provide a new direction for the diagnosis and treatment of HUA and gout.

## Conclusions

In recent years, the application of molecular tools to the study of the human gut microbiota has led to dramatic changes in the abundance of certain bacterial groups. Our research revealed that lower community richness and diversity were altered during the process of gout or HUA. In addition, the community richness or diversity decreased more when acute gout flared. We also observed that metagenomic species changed significantly during different stages of hyperuricemia. A notable limitation of this study is the insufficient granularity of the dietary assessment, which precludes a detailed characterization of habitual intake, particularly concerning specific prebiotic fiber sources like garlic, onion, leek, and asparagus. Comprehensive dietary assessment methodologies should be taken to elucidate the complex interplay between specific dietary components, microbial function, and hyperuricemic pathogenesis in our future investigations. Although our results revealed that dysbiosis of the gut microbiome was associated with increased SUA and may be variable in different stages of gout, the functional implications of these microbial shifts—such as altered short-chain fatty acid (SCFA) production—remain unexamined. Future studies on larger longitudinal cohorts, together with animal model experiments, and functional analyses are needed to validate our findings toward a better understanding of the underlying mechanisms of the gut microbiota in different stages of gout.

## Data Availability

The datasets presented in this study can be found in online repositories. The names of the repository/repositories and accession number(s) can be found in the article/**Supplementary Material**.

## Supplementary material

Supplementary Figure 1 Histogram of the intestinal flora at the phylum and genus levels.

Supplementary Figure 2 The altered species of the gut microbiota between hyperuricemic patients and healthy controls via LDA analysis and a cladogram generated via LEfSe analysis.

Supplementary Figure 3 The altered species of the gut microbiota between asymptomatic HUA patients and gout patients according to LEfSe analysis via LDA analysis and a cladogram.

Supplementary Figure 4 The altered species of the gut microbiota based on LDA analysis and Cladogram according to LEfSe analysis between the resolution gout group and acute gout group.

Supplementary Figure 5 Heatmap based on Spearman correlations between age, BMI, blood pressure, ALT, AST, FDG, blood lipids, serum uric acid, and creatinine levels and the gut microbiota at the phylum level with a relative abundance >0.1%. *Statistical significance P < 0.05.

Supplementary Figure 6 Heatmap based on Spearman correlations between age, BMI, blood pressure, ALT, AST, FDG, blood lipids, serum uric acid, and creatinine levels and the gut microbiota at the genus level with a relative abundance >0.1%. *Statistical significance P < 0.05. **Statistical significance P < 0.01.
