# Supplementary material for: The gut microbiome: a vital link to hyperuricemia, gout and acute flares?
Source: Front Endocrinol (Lausanne). 2025 Aug 8;16:1643566. doi: 10.3389/fendo.2025.1643566 (PMC12370519; doi:10.3389/fendo.2025.1643566)
Supplement: Supplementary Figure 1 — Histogram of the intestinal flora at the phylum and genus levels. [file Image1.pdf]

Supplementary material

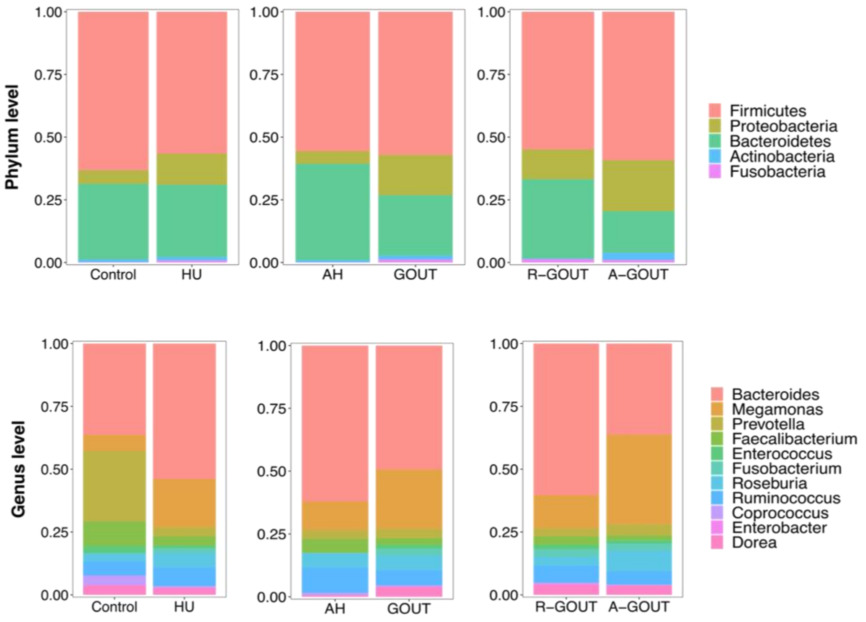

Supplementary Figure S1. Histogram of the intestinal flora at the phylum and genus levels

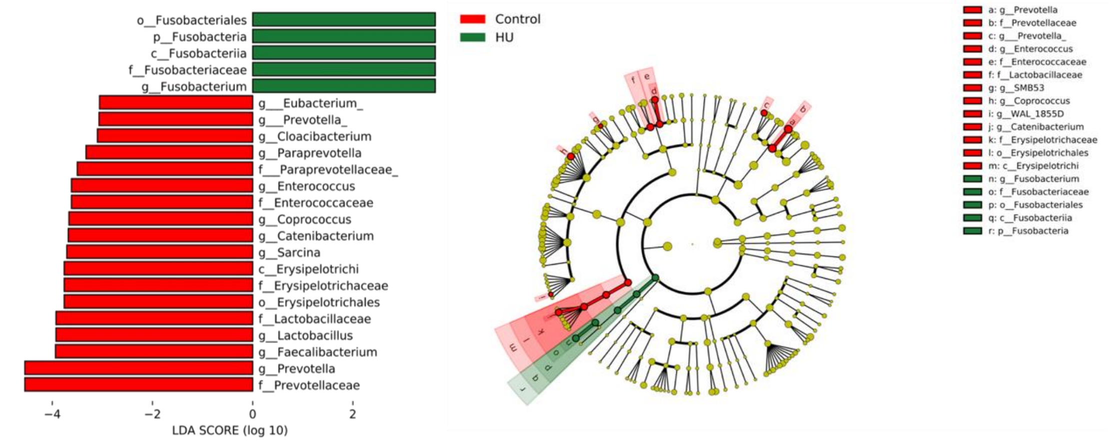

Supplementary Figure S2. The altered species of the gut microbiota between hyperuricemic patients and healthy controls via LDA analysis and a cladogram generated via LEfSe analysis.

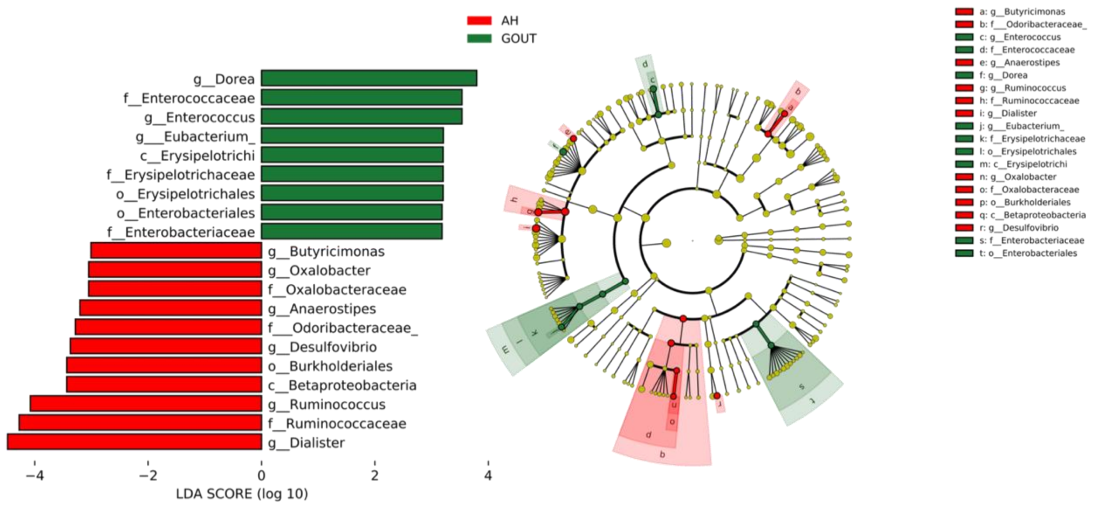

Supplementary Figure S3. The altered species of the gut microbiota between asymptomatic HUA patients and gout patients according to LEfSe analysis via LDA analysis and a cladogram.

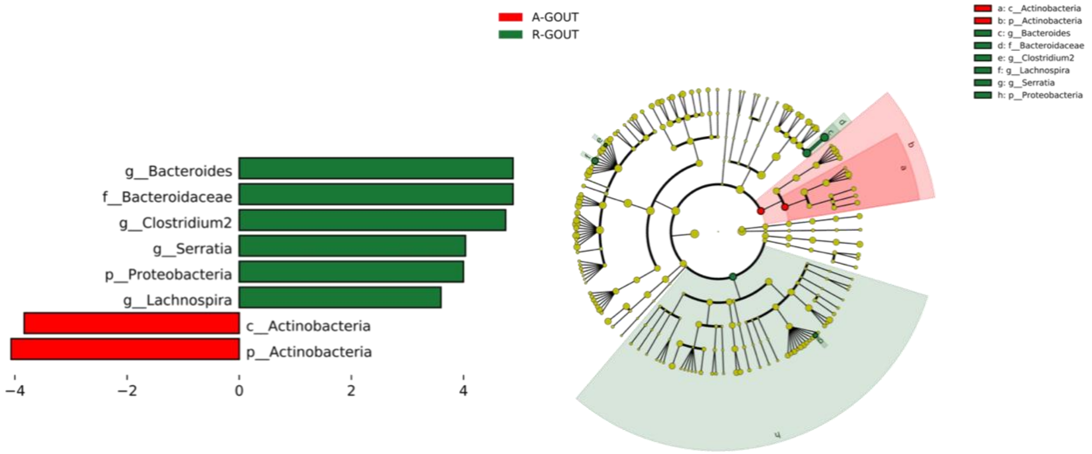

Supplementary Figure S4. The altered species of the gut microbiota based on LDA analysis and Cladogram according to LEfSe analysis between the resolution gout group and acute gout group.

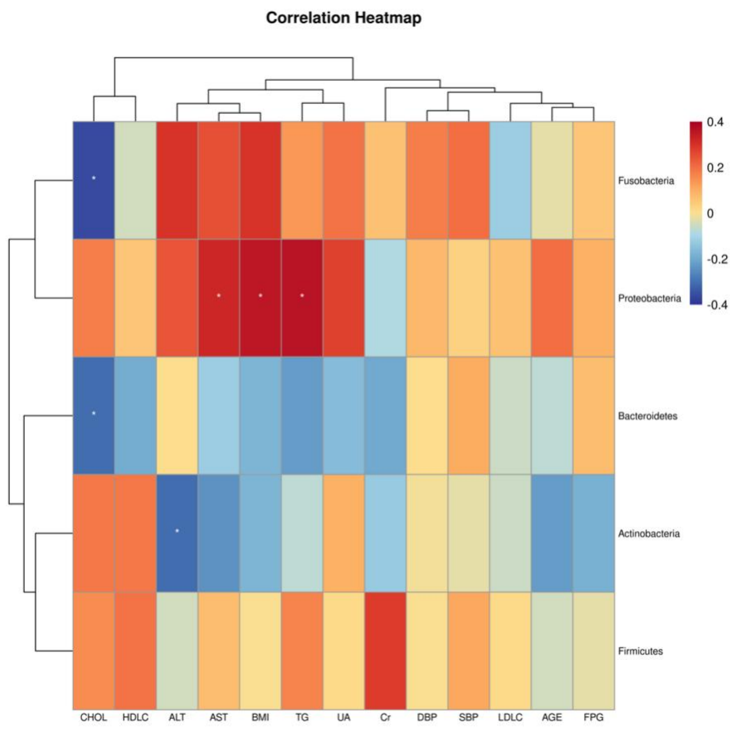

Supplementary Figure S5. Heatmap based on Spearman correlations between age, BMI, blood pressure, ALT, AST, FDG, blood lipids, serum uric acid, and creatinine levels and the gut microbiota at the phylum level with a relative abundance >0.1%. *Statistical significance P < 0.05.

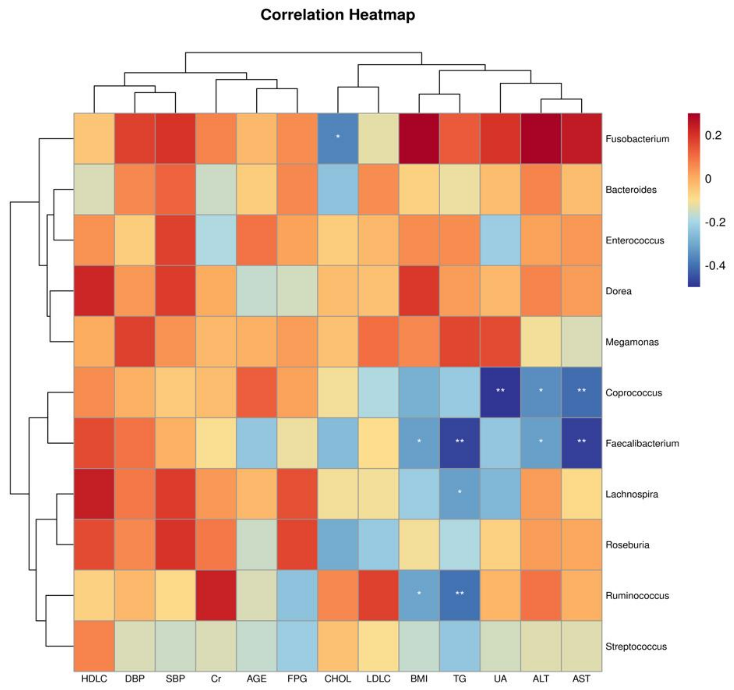

Supplementary Figure S6. Heatmap based on Spearman correlations between age, BMI, blood pressure, ALT, AST, FDG, blood lipids, serum uric acid, and creatinine levels and the gut microbiota at the genus level with a relative abundance >0.1%. *Statistical significance P < 0.05. **Statistical significance P < 0.01.
